# Efficacy of Metal Stents Versus Plastic Stents for Treatment of Walled‐Off Pancreatic Necrosis: A Systematic Review and Meta‐Analysis

**DOI:** 10.1002/jgh3.70109

**Published:** 2025-02-03

**Authors:** Yousaf Zafar, Muhammad Umer Sohail, Zainab Siddiqua Ibrahim, Ruqiat Masooma Batool, Ifrah Ansari, Syed Zaeem Ahmed, Muhammad Saad, Eliza Aisha, Saad Ahmed Waqas, Muhammad Ovais Sohail, Faisal Bukeirat, Shou Jiang Tang, Raheel Ahmed

**Affiliations:** ^1^ Department of Internal Medicine University of Mississippi Medical Center Jackson USA; ^2^ Department of Medicine Dow University of Health Sciences Karachi Pakistan; ^3^ Department of Medicine Conemaugh Memorial Medical Center Johnstown USA; ^4^ Department of Digestive Diseases University of Mississippi Medical Center Jackson USA; ^5^ National Heart and Lung Institute Imperial College London London UK

**Keywords:** metal stents, plastic stents, walled‐off pancreatic necrosis

## Abstract

**Background:**

Walled‐off necrosis (WON) is a potentially fatal condition best treated endoscopically with metal or plastic stents. This study compares the clinical outcomes of these stents.

**Methods:**

PubMed and Cochrane were searched for trials comparing metal and plastic stents for WON. Primary outcomes were clinical and technical success.

**Results:**

Seven studies with 230 metal stent patients and 226 plastic stent patients were included. Metal stents showed significantly shorter procedure times (SMD ‐0.80, 95% CI: ‐1.25 to −0.34), better 4‐week clinical success (OR 1.94, 95% CI: 1.00 to 3.77), and higher procedure costs (SMD 1.38, 95% CI: 0.56 to 2.20). No significant differences were observed in hospital stay (SMD ‐0.05, 95% CI: ‐0.35 to 0.25), technical success (OR 1.45, 95% CI: 0.22 to 9.43), clinical success (OR 1.13, 95% CI: 0.54 to 2.39), interventions (SMD ‐0.02, 95% CI: ‐0.34 to 0.29), need for necrosectomy (RR 1.10, 95% CI: 0.59 to 2.04), necrosectomy sessions (SMD 0.35, 95% CI: ‐0.42 to 1.11), need for percutaneous drainage (RR 0.82, 95% CI: 0.36 to 1.85), stent migration (RR 0.88, 95% CI: 0.29 to 2.66), bleeding (RR 0.97, 95% CI: 0.53 to 1.75), WON recurrence (RR 1.66, 95% CI: 0.70 to 3.92), treatment failure (death) (RR 0.75, 95% CI: 0.37 to 1.53), disconnected pancreatic duct (RR 0.93, 95% CI: 0.79 to 1.11), and total cost (SMD ‐0.02, 95% CI: ‐0.29 to 0.26).

**Conclusion:**

Metal stents offer shorter procedure time and better 4‐week clinical success, although at a higher cost, with most clinical outcomes showing no significant differences between stent types.

## 1 Introduction

Infected walled‐off necrosis (WON) is a severe complication of acute pancreatitis, characterized by encapsulated necrotic tissue with high morbidity and mortality rates [1, 2]. Endoscopic interventions have increasingly supplanted traditional surgical methods for treating WON, owing to their association with a reduced risk of new‐onset multi‐organ failure, decreased overall healthcare costs, and shorter hospital stays [3, 4, 5, 6]. Endoscopic ultrasound (EUS)‐guided drainage, utilizing either metal or plastic stents, represents the forefront of these minimally invasive interventions [3, 7, 8, 9, 10, 11, 12].

Lumen‐apposing metal stents (LAMS) are increasingly being used for the endoscopic drainage of pancreatic and peripancreatic necrotic fluid collections. Their large diameter reduces the risk of stent occlusion, offers the possibility of direct endoscopic necrosectomy (DEN), and facilitates efficient drainage of necrotic material, potentially reducing the need for additional interventions and necrosectomies [13, 14, 15, 16]. However, LAMS are associated with higher costs and increased stent‐related complications if not removed within 3 weeks [17, 18].

Despite the growing use of LAMS, guidelines for their use compared with plastic stents remain ambiguous. The European Society of Gastrointestinal Endoscopy suggests both LAMS and plastic stents as viable options, whereas the Asian consensus guidelines recommend LAMS usage only within clinical trials [19, 20]. Contrarily, the American Gastroenterological Association favors LAMS [21].

A recent meta‐analysis by Bang et al. found no clinical superiority of LAMS over plastic stents [22]. Our study aims to expand on these findings by incorporating four additional trials thus providing a more comprehensive assessment with double the sample size and broader outcome measures.

## 2 Methods

This systematic review and meta‐analysis adhered to the methodological standards delineated in the Cochrane Handbook for Systematic Reviews of Interventions and is reported in conformity with the Preferred Reporting Items for Systematic Reviews and Meta‐Analyses (PRISMA) guidelines [23, 24].

### 2.1 Eligibility Criteria and Outcomes

Eligible studies were restricted to peer‐reviewed articles published in English and involving human participants. Inclusion criteria required the following: (1) randomized controlled trials or their post hoc analyses; (2) studies involving patients aged 18 years and over diagnosed with WON; and (3) comparisons of EUS‐guided drainage using metal stents versus plastic stents. Exclusion criteria encompassed the following: (1) studies that did not involve human participants; (2) single‐arm studies; (3) publications categorized as case reports, reviews, or observational studies.

The primary outcomes assessed were technical success and clinical success. Secondary outcomes included the duration of hospital stay, total procedure time, the number of interventions required, the need for DEN, total necrosectomy sessions, the requirement for percutaneous catheter drainage, the incidence of stent migration, bleeding episodes, recurrence of WON, treatment failure (measured as death), the incidence of a disconnected pancreatic duct, and the costs associated with the procedure and overall treatment.

### 2.2 Data Sources and Search Strategy

A comprehensive electronic search was conducted in PubMed and the Cochrane Central Register of Controlled Trials from inception to June 26, 2024. The detailed search strategy is outlined in Table S1. Articles identified through the systematic search were imported into rayyan.ai for duplicate removal [25]. The selection process was conducted in two stages: an initial screening based on titles and abstracts, followed by a full‐text review using predefined eligibility criteria. Both stages were independently performed by two reviewers (MS and SZA) with any discrepancies resolved through consultation with a third reviewer (MUS).

### 2.3 Data Extraction and Quality Assessment

Two independent reviewers (MS and EA) carried out data extraction and quality assessment of the included studies. Any disagreements were resolved through discussion. Extracted data included baseline characteristics, trial demographics, and outcome measures, which were documented in an Excel spreadsheet. The quality of the included studies was assessed using the Cochrane risk of bias tool [26]. To ensure the accuracy and reliability of the extracted data, all authors collectively reviewed the information.

### 2.4 Statistical Analysis

For continuous outcomes, the mean change from baseline was used to determine effect sizes. If only baseline and endpoint data were available, the mean change and its standard deviation (SD) were calculated using the formula: [23, 27].

ΔMean = Mean endpoint—Mean baseline.

ΔSD = √ SD2 baseline + SD2 endpoint − (2× r × SD baseline× SD endpoint).

where r represents the correlation coefficient. A conservative value of r = 0.7 was used to minimize estimation bias [28]. For outcome measurements reported as median (interquartile range), the Box–Cox method was applied to transform these values to mean (SD) [29]. Effect sizes were aggregated using a random‐effects model to compute standardized mean differences (SMDs), risk ratios (RRs) or odds ratios (OR) with their 95% confidence intervals (CIs). Forest plots were created for visual representation, and funnel plots were used to assess potential publication bias. The Higgins *I* ^2^ statistic was employed to evaluate heterogeneity across studies, with *I* ^2^ values of 25%, 50%, and 75% considered benchmarks for low, medium, and high heterogeneities, respectively. Statistical significance was determined with a *p*‐value of < 0.05. Sensitivity analyses were conducted to assess the robustness of the results by excluding individual studies to determine their impact on the overall findings. All statistical analyses were performed using Review Manager (Version 5.4, Copenhagen: The Nordic Cochrane Centre, The Cochrane Collaboration, 2020).

## 3 Results

### 3.1 Description of Included Studies and Patient Characteristics

A search of PubMed and Cochrane databases initially identified 129 results. Following the removal of duplicates, 80 studies were screened. Of these, 73 studies were excluded based on title and abstract screening, leaving 7 studies for full‐text review. These 7 studies, involving a total of 456 patients, were included in the meta‐analysis [7, 8, 9, 10, 11, 12, 17]. Although our primary inclusion criterion focused on RCTs with direct randomization between metal and plastic stents, we included Boxhoorn et al. due to its similar methodology as other trials. This study draws patients from two multicenter RCTs with consistent designs and patient criteria: the AXIOMA trial, which used metal stents (LAMS), and the TENSION trial, which used plastic stents. This setup allows for a reliable comparison between stent types, despite the lack of randomization within a single trial. The PRISMA flow chart summarizes the search and trial selection (Figure S1).

The average age for patients was 50.68 and 53.14 years in the metal and plastic stent groups, respectively. The baseline characteristics for patients in each study are presented in Table 1. Follow‐up time ranged from 3 to 12 months. The study time span included dates from 2017 to 2023, covering various geographical locations like the USA, India, the Netherlands, Spain, Denmark, and Korea. All studies included patients over the age of 18 presenting with WON as a complication of acute pancreatitis. One study included only those presenting with infected WON [8]. All included trials were at a moderate risk‐of‐bias (Figure S2).

**TABLE 1:** Summary of Baseline Characteristics in Studies Comparing Metal and Plastic Stents for Treatment of Walled‐Off Pancreatic Necrosis.

		Kakadiya 2023	Gornals 2024	Bang 2018	Karstensen 2022	Boxhoorn 2022	Koduri 2024	Moon 2024
Study characteristics								
Study Design		RCT	RCT	RCT	RCT	RCT + Prospective	RCT	RCT
Study Site		India	Spain	USA	Denmark	Netherlands	India	Korea
Number of Patients		48	64	60	42	104	92	46
Follow‐up, months		3	12	6	3	6	6	3
Patients characteristics								
Age, mean (SD)	Metal	35.4 (11.8)	60.0 (13.5)	55.8 (15.6)	59.4 (13.0)	59.0 (13.0)	34.9 (12.4)	51.2 (17.0)
	Plastic	38.2 (12.7)	62.0 (15.4)	60.3 (13.0)	61.1 (14.3)	63.0 (14.0)	36.8 (11.1)	51.0 (16.2)
Males, *n* (%)	Metal	18.0 (75.0)	25.0 (76.0)	20.0 (64.5)	15.0 (75.0)	33.0 (62.0)	38.0 (82.6)	16.0 (69.6)
	Plastic	20.0 (83.3)	20.0 (64.0)	16.0 (55.2)	17 (77.3)	34.0 (67.0)	38.0 (82.6)	11.0 (47.8)
Necrotic collection characteristics								
WON size [cm], mean (SD)	Metal	13.7 (3.5)	11.2 (4.7)	10.2 (4.6)	26.4 (10.1)	NA	14.2 (4.7)	8.9 (5.6)
	Plastic	11.4 (3.0)	11.5 (3.5)	10.7 (6.8)	23.4 (6.7)	NA	15.0 (15.8)	7.5 (3.4)
Infected necrosis, *n* (%)	Metal	9.0 (37.5)	13.0 (40.0)	27.0 (87.1)	17.0 (77.3)	19.0 (36.0)	31.0 (67.4)	23.0 (100.0)
	Plastic	3.0 (12.5)	13.0 (42.0)	26.0 (89.7)	19.0 (95.0)	23.0 (45.0)	33.0 (71.7)	23.0 (100.0)
White Cell Count [×10^9 cells/L], mean (SD)	Metal	NA	9.2 (4.1)	11.2 (7.0)	13.2 (7.0)	16.1 (8.4)	9.1 (5.4)	0.008 (0.003)
	Plastic	NA	8.4 (3.0)	11.3 (6.5)	11.5 (5.2)	13.3 (6.1)	8.5 (3.9)	0.008 (0.004)
WON location, *n* (%)								
Head/Uncinate	Metal	NA	11.0 (36.0)	9.0 (29.0)	6.0 (31.5)	NA	12.0 (26.1)	2.0 (8.7)
Body/Tail	Metal	NA	19.0 (63.0)	22.0 (71.0)	12.0 (63.1)	NA	34.0 (65.2)	19.0 (82.6)
Head/Uncinate	Plastic	NA	10.0 (32.0)	6.0 (20.7)	5.0 (22.7)	NA	11.0 (23.9)	2.0 (8.7)
Body/Tail	Plastic	NA	21.0 (67.0)	23.0 (79.3)	11.0 (55.0)	NA	35.0 (76.1)	20.0 (87.0)
Disease severity								
Systemic Inflammatory Response Syndrome, *n* (%)	Metal	10.0 (41.7)	NA	9.0 (29.0)	12.0 (60.0)	47.0 (89.0)	37.0 (80.4)	NA
	Plastic	4.0 (16.7%)	NA	13.0 (44.8)	15 (68.2)	33.0 (65.0)	35.0 (70.1)	NA
CRP [mg/L], mean (SD)	Metal	75.8 (71.9)	129.8 (79.9)	NA	131.2 (151.8)	238.5 (137.9)	43.6 (60.7)	NA
	Plastic	52.2 (64.5)	101.4 (69.7)	NA	140.9 (86.9)	177.2 (114.4)	40.0 (43.9)	NA
ICU Admissions, *n* (%)	Metal	NA	6.0 (18.0)	10.0 (32.3)	9.0 (45.0)	14.0 (26.0)	38.0 (82.6)	NA
	Plastic	NA	6.0 (19.0)	10.0 (34.5)	5.0 (22.7)	21.0 (41.0)	34.0 (73.9)	NA
Organ Failure, *n* (%)	Metal	1.0 (4.2)	7.0 (25.0)	2.0 (6.5)	6.0 (30.0)	12.0 (23.0)	NA	NA
	Plastic	1.0 (4.2)	7.0 (26.9)	4.0 (13.8)	3.0 (13.6)	13.0 (25.0)	NA	NA
Etiology of acute pancreatitis, *n* (%)								
Alcohol	Metal	9.0 (37.5)	9.0 (28.0)	9.0 (29.0)	5.0 (25.0)	NA	6.0 (14.0)	8.0 (34.8)
	Plastic	10.0 (41.7)	8.0 (25.0)	5.0 (17.2)	5.0 (22.7)	NA	10.0 (21.0)	9.0 (39.1)
Gallstones	Metal	10.0 (41.7)	NA	6.0 (19.4)	14.0 (70.0)	NA	NA	0.0 (0.0)
	Plastic	10.0 (41.7)	NA	10.0 (34.5)	10 0.0 (45.5)	NA	NA	1.0 (4.3)
Trauma	Metal	0.0 (0.0)	NA	1.0 (3.2)	NA	NA	NA	NA
	Plastic	1.0 (4.2)	NA	1.0 (3.4)	NA	NA	NA	NA
Type of stent used	Metal	BFCSEM stents, or Hot AXIOS stents(LAMS)	Hot AXIOS Stents(LAMS)	Hot AXIOS Stents(LAMS)	Hot AXIOS Stents(LAMS)	Hot AXIOS Stents(LAMS)	BFCSEM stents	Hot SPAXUS stent(LAMS)
	Plastic	Double‐pigtail plastic stents	Double‐pigtail plastic stents	Double‐pigtail plastic stents	Double‐pigtail plastic stents	Double‐pigtail plastic stents	Double‐pigtail plastic stents	Double‐pigtail plastic stents

Abbreviations: BFCSEM: Biflanged fully‐covered self‐expanding metallic; CRP: C‐reactive protein; ICU: intensive care unit; LAMS: lumen‐apposing metal stents.

### 3.2 Description of Interventions Used

Each study made use of EUS‐guided WON drainage. The 7‐Fr double pigtail plastic stents were used in all plastic stent groups. In the LAMS groups, almost all studies used the HOT Axios stents [9, 10, 11, 17], except Kakadiya et al., Koduri et al., and Moon et al., who used Biflanged fully‐covered self‐expanding metallic (BFCSEM) stents and HOT Axios, BFCSEM, and Hot SPAXUS metal stents, respectively.

### 3.3 Meta‐Analyses Results

#### 3.3.1 Procedure Time

**FIGURE 1:**
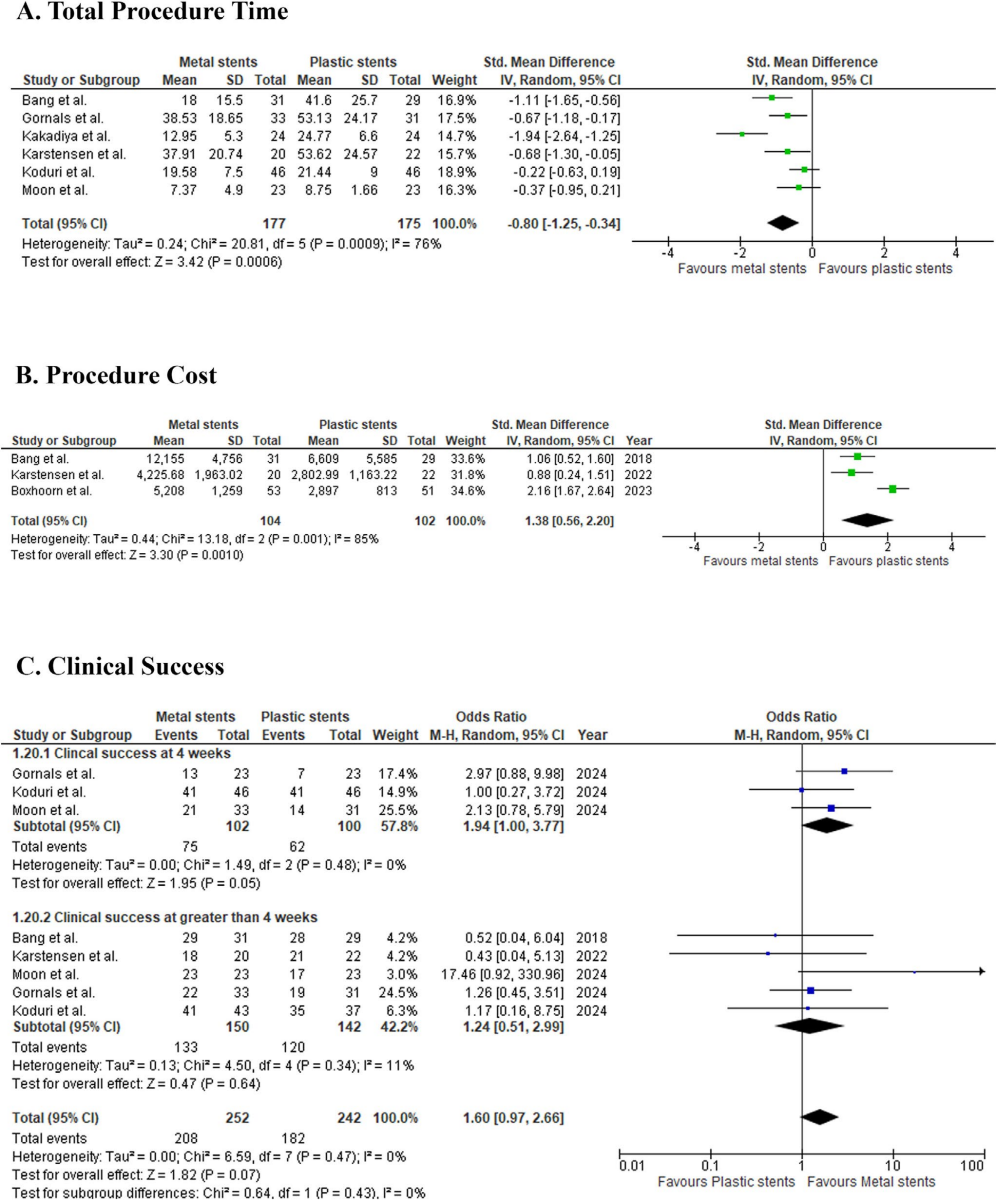
The effect of metal stent compared with plastic stent on (A) total procedure time, (B) procedure cost, and (C) clinical success in walled‐off pancreatic necrosis patients.

The analysis of six studies involving 352 patients demonstrated that metal stents significantly reduced the total procedure time compared with plastic stents. The pooled SMD was −0.80 (95% CI: −1.25 to −0.34; *p* = 0.0006; *I* ^2^ = 76%; Figure 1A), indicating that metal stents required less time. Sensitivity analysis, conducted by excluding the study by Kakadiya et al. reduced heterogeneity to *I* ^2^ = 44%, but the effect size remained consistent (SMD: −0.59; 95% CI: −0.90 to −0.27; *p* = 0.0003; Figure S3).

#### 3.3.2 Procedure Cost

In the comparison of procedural costs, three studies encompassing 206 patients revealed that metal stents were associated with significantly higher costs. The pooled SMD was 1.38 (95% CI: 0.56 to 2.20; *p* = 0.0010; *I* ^2^ = 85%; Figure 1B). Sensitivity analysis, excluding Boxhoorn et al., lowered heterogeneity (*I* ^2^ = 0%) but did not alter the effect size meaningfully (SMD: 0.98; 95% CI: 0.57 to 1.39; *p* < 0.00001; Figure S4).

#### 3.3.3 Clinical Success

Clinical success was evaluated over two periods: short‐term (4 weeks) and long‐term (more than 4 weeks). Three studies with 202 patients reported that metal stents had superior clinical success at 4 weeks, with a pooled OR of 1.94 (95% CI: 1.00 to 3.77; *p* = 0.05; *I* ^2^ = 0%; Figure 1C). In contrast, five studies involving 292 patients found no significant difference between stent types beyond 4 weeks (OR: 1.24; 95% CI: 0.51 to 2.99; *p* = 0.64; *I* ^2^ = 11%; Figure 1C). The overall clinical success across all studies was also comparable (OR: 1.60; 95% CI: 0.97 to 2.66; *p* = 0.43; *I* ^2^ = 0%; Figure 1C).

#### 3.3.4 Hospital Stay

**FIGURE 2:**
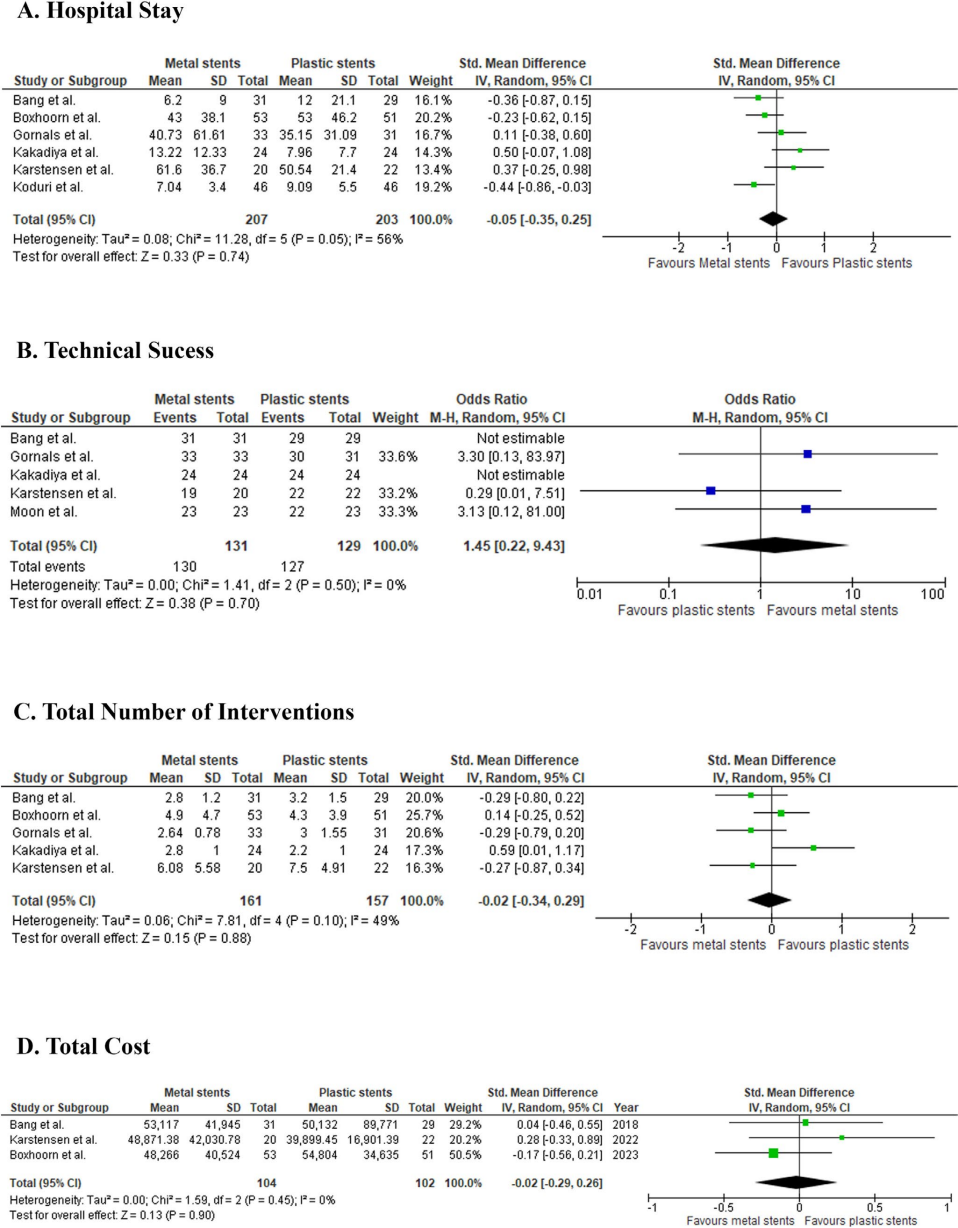
The effect of metal stent compared with plastic stent on (A) hospital stay, (B) technical success, (C) total number of interventions, and (D) total cost in walled‐off pancreatic necrosis patients.

The duration of hospital stay was compared across six studies with 410 patients. The pooled SMD was −0.05 (95% CI: −0.35 to 0.25; *p* = 0.74; *I* ^2^ = 56%; Figure 2A), indicating no meaningful difference between the two stent types. Sensitivity analysis, excluding the study by Kakadiya et al., reduced heterogeneity (*I* ^2^ = 39%) without affecting the effect size (SMD: −0.15; 95% CI: −0.42 to 0.11; *p* = 0.26; Figure S5).

#### 3.3.5 Technical Success

Five studies, comprising a total of 260 patients, compared technical success between patients treated with metal stents and those treated with plastic stents. The pooled OR was 1.45 (95% CI: 0.22 to 9.43; *p* = 0.70, *I* ^2^ = 0%; Figure 2B), indicating no significant difference in technical success between the metal and plastic stent groups.

#### 3.3.6 Total Number of Interventions

A comparison of the total number of interventions, involving 318 patients from five studies, revealed no significant difference between the two stent groups (SMD: −0.02; 95% CI: −0.34 to 0.29; *p* = 0.88; *I* ^2^ = 49%; Figure 2C).

#### 3.3.7 Total Treatment Cost

Three studies with 206 patients assessed the total cost of treatment. The pooled SMD was −0.02 (95% CI: −0.29 to 0.26; *p* = 0.90; *I* ^2^ = 0%; Figure 2D), showing no significant cost difference between the two stent types when considering overall treatment expenses.

#### 3.3.8 Need for DEN

**FIGURE 3:**
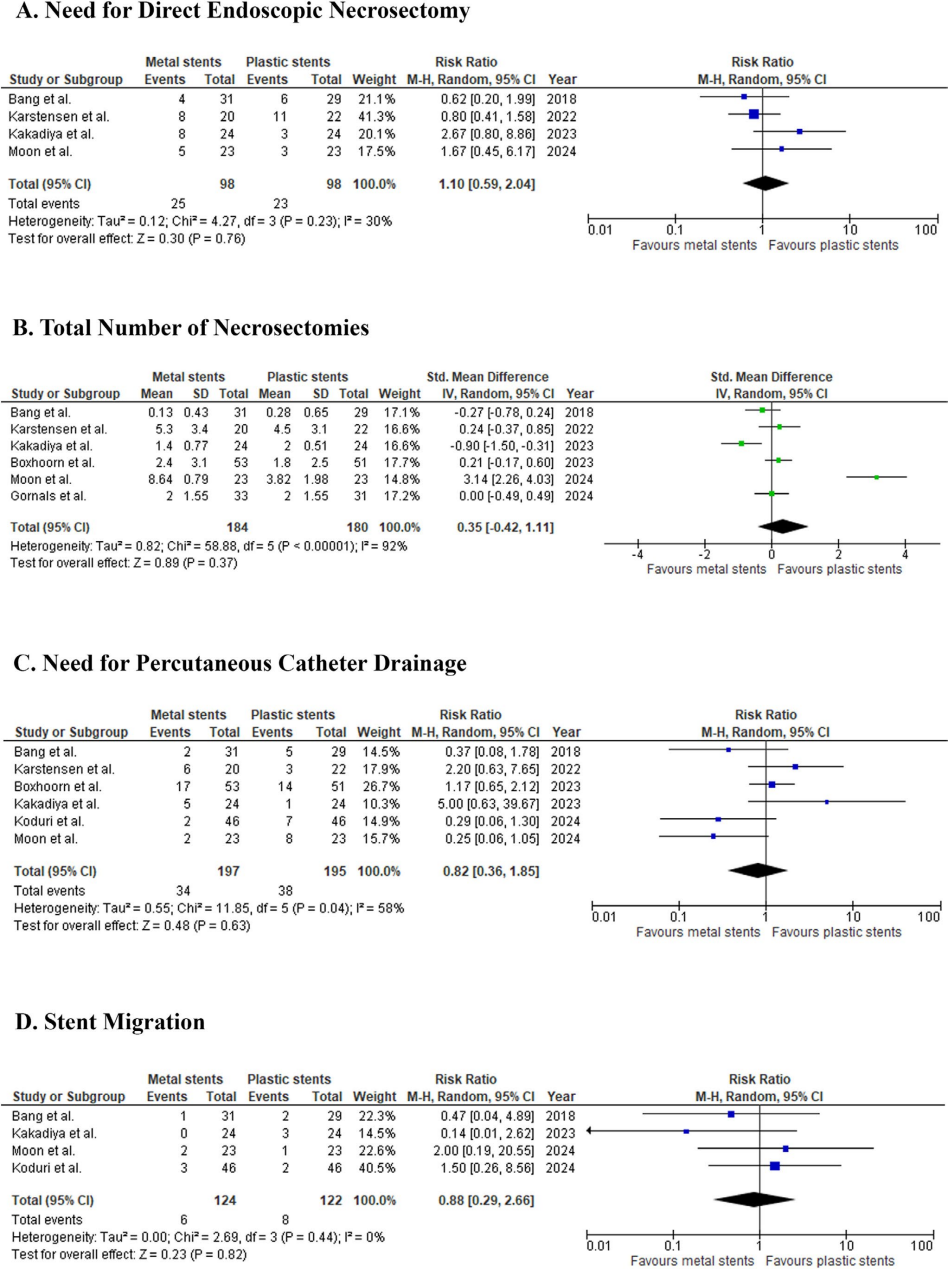
The effect of metal stent compared with plastic stent on (A) the need for direct endoscopic necrosectomy, (B) the total number of necrosectomies, (C) the need for percutaneous catheter drainage, and (D) stent migration in walled‐off pancreatic necrosis patients.

The requirement for DEN was compared across four studies involving 196 patients. The pooled RR was 1.10 (95% CI: 0.59 to 2.04; *p* = 0.76; *I* ^2^ = 30%; Figure 3A), indicating no significant difference between metal and plastic stents.

#### 3.3.9 Number of Necrosectomy Sessions

The total number of necrosectomy sessions was examined across six studies with 364 patients. The pooled SMD was 0.35 (95% CI: −0.42 to 1.11; *p* = 0.37; *I* ^2^ = 92%; Figure 3B), indicating no significant difference between the two stent types. Sensitivity analysis, excluding Moon et al. and Kakadiya et al., reduced heterogeneity (I^2^ = 0%) but did not alter the results (SMD: 0.06; 95% CI: −0.18 to 0.30; *p* = 0.63; Figure S6).

#### 3.3.10 Need for Percutaneous Catheter Drainage

Six studies with 392 patients assessed the need for percutaneous catheter drainage, with the pooled RR calculated at 0.82 (95% CI: 0.36 to 1.85; *p* = 0.63; *I* ^2^ = 58%; Figure 3C). Sensitivity analysis, excluding Moon et al. and Koduri et al., reduced heterogeneity (*I* ^2^ = 38%) but did not alter the effect size significantly (RR: 1.31; 95% CI: 0.61 to 2.84; *p* = 0.49; Figure S7).

#### 3.3.11 Incidence of Stent Migration

The incidence of stent migration was reported in four studies involving 246 patients. The pooled RR was 0.88 (95% CI: 0.29 to 2.66; *p* = 0.82; *I* ^2^ = 0%; Figure 3D), indicating no significant difference between the two stent types regarding stent migration.

#### 3.3.12 Disconnected Pancreatic Duct

**FIGURE 4:**
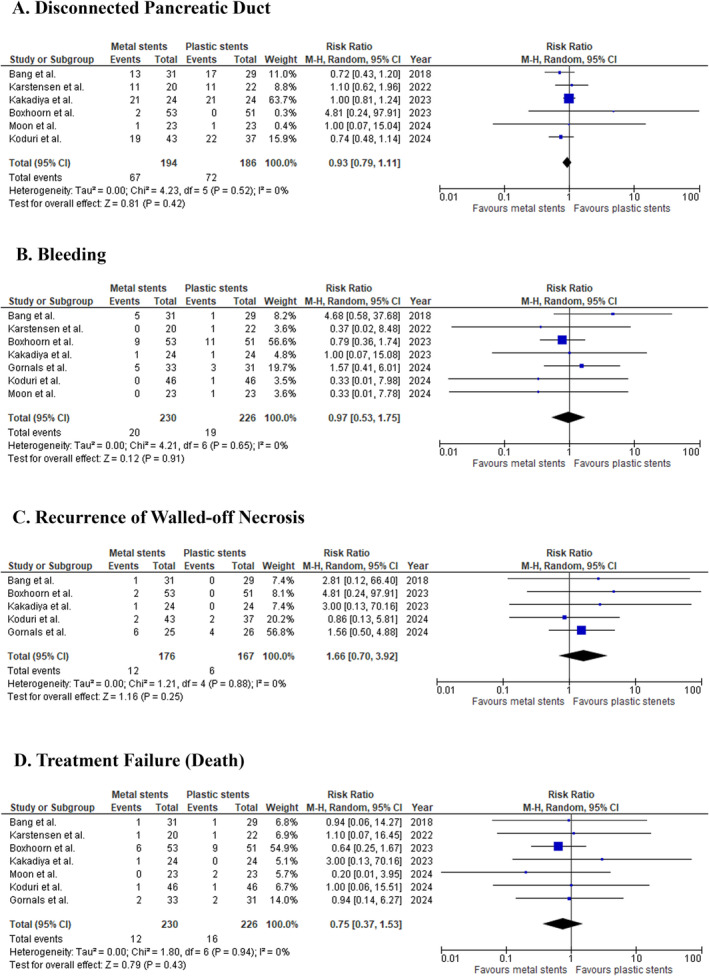
The effect of metal stent compared with plastic stent on (A) disconnected pancreatic duct, (B) bleeding, (C) recurrence of walled‐off necrosis, and (D) treatment failure (resulting in death) in walled‐off pancreatic necrosis patients.

Six studies with 380 patients compared the incidence of a disconnected pancreatic duct. The pooled RR was 0.93 (95% CI: 0.79 to 1.11; *p* = 0.42; *I* ^2^ = 0%; Figure 4A), showing no significant difference between metal and plastic stents.

#### 3.3.13 Bleeding

Seven studies, comprising a total of 456 patients, compared the incidence of bleeding between patients treated with metal stents and those treated with plastic stents. The pooled RR was 0.97 (95% CI: 0.53 to 1.75; *p* = 0.91, *I* ^2^ = 0%; Figure 4B), indicating no significant difference in the incidence of bleeding between the metal and plastic stent groups.

#### 3.3.14 Recurrence of WON

Five studies with 343 patients evaluated the recurrence of WON. The pooled RR was 1.66 (95% CI: 0.70 to 3.92; *p* = 0.25; *I* ^2^ = 0%; Figure 4C), indicating no significant difference between metal and plastic stents in preventing recurrence.

#### 3.3.15 Treatment Failure (Mortality)

The analysis of mortality rates across seven studies with 456 patients showed no significant difference between the two stent types (RR: 0.75; 95% CI: 0.37 to 1.53; *p* = 0.43; *I* ^2^ = 0%; Figure 4D).

## 4 Discussion

In this meta‐analysis, spanning 456 patients, comparing the efficacy of metal stents versus plastic stents for the transmural drainage of WON, we report several key findings. Our analysis revealed that although procedural costs were significantly lower with plastic stents, the procedure time was significantly longer than with the metal stents. Moreover, significantly greater short‐term clinical success was observed with metal stents.

Our meta‐analysis builds upon prior assessments of LAMS for WON management by focusing exclusively on RCTs, ensuring enhanced methodological rigor and clinical applicability. Previous studies pooled data from tertiary‐care centers and observational studies [30, 31, 32] —introducing biases and limiting generalizability. Additionally, we address critical gaps unexplored in earlier studies, including comprehensive evaluations of DEN [32] and cost analyses [33], both vital for clinical and economic evaluation. Our larger sample size also reduces the risk of type II error, a common limitation in prior studies with smaller, heterogeneous samples [22], enabling a more accurate comparison between LAMS and plastic stents.

Interventions, such as drainage or debridement, are considered for symptomatic or infected WON only [34, 35, 36], with some reports favoring LAMS over plastic stents [21]. While our meta‐analysis found no significant differences in long‐term clinical success between the stents, borderline significant results favoring metal stents for short‐term clinical success were observed. This improved short‐term efficacy of metal stents over plastic stents aligns with existing literature, which shows that LAMS can achieve rapid clinical success due to their wider diameter, leading to faster WON resolution [30, 33, 37, 38, 39]. Studies also emphasize the need to remove LAMS after 4 weeks due to an increased risk of adverse events as their safety declines with prolonged use [17, 18, 32, 39, 40, 41]. Our finding of metal stent superiority over plastic stents for short‐term clinical success suggests that the use of metal stents may be ideal in older, critically ill, or comorbid patients, particularly those with prior failed drainage [7, 11, 22, 41, 42]. The enhanced short‐term efficacy of metal stents may provide a more proactive, aggressive approach crucial for these patients [42]. The shorter procedural duration and improved safety associated with short‐term LAMS use could also help prevent complications in this vulnerable group. Additionally, patients with more complex WON, characterized by larger size or greater solid content, may benefit from LAMS [17, 43, 44], which enables quicker and more thorough drainage, thereby reducing the risk of worsening infection, tissue hyperplasia, and stent entrapment due to slow or incomplete drainage [17, 39, 45]. However, careful patient follow‐up is essential when assessing long‐term success. Prolonged use in patients at higher risk of bleeding, such as those with pseudoaneurysms near the WON, should be particularly avoided [17, 43]. The similar long‐term efficacy of both stents is consistent with some research findings which suggest that WON gradually liquefies and shrinks over time, rendering both stents comparable in the long‐term [34, 46, 47]. Therefore, metal stents should be optimized for short‐term use, especially in cases with significant debris [34], with further research needed to develop ideal strategies and enhance long‐term surveillance [48].

We also identified a need for greater standardization in clinical success assessment across trials. Substantial heterogeneity in trial definitions and follow‐up times for long‐term clinical success may have confounded true effects. Follow‐up in trials varied from 8 weeks to 3,4 or 6 months [7, 8, 9, 11, 17]. Assessing short‐term clinical success at 4 weeks provided a more standardized outcome, but this was reported separately in only three trials [7, 8, 11]. The definition of clinical success (radiologic vs. symptomatic resolution) and the criteria for DEN also varied between studies, leading to incohesive results. This lack of consensus resulted in differing methods of accounting for outcomes such as clinical success and the need for DEN in the various studies, thus causing a lack of standardization. Moreover, some studies reported better baseline characteristics in their plastic groups, which may have led to more favorable outcomes. These differences included variations in WON sizes and contents [8, 9, 11], and worse preprocedural clinical presentation in metal stent patients, such as a higher rate of systemic inflammatory response syndrome (SIRS), elevated C reactive protein (CRP) levels [10], and a greater need for preprocedural ICU care [9], potentially skewing results. Lastly, trials used different lengths of metal stents, including 10‐, 15‐, or 20‐mm LAMS or 15‐mm BFMS. These discrepancies could introduce statistical noise, complicating the formation of definitive conclusions.

The recommended treatment for WON involves an endoscopic step‐up approach, starting with EUS‐guided drainage, followed by additional interventions if needed [48]. However, some trials deviated from standard protocols. Karstensen et al., a major outlier, used nasocystic catheters with irrigation and coaxial plastic stents in the LAMS group at the index procedure instead of simple stent placement; they also employed the multigate technique and a weekly tract dilation protocol for the plastic group. Bang et al. also used the multigate technique, as well as nasocystic catheters at index along with Boxhoorn et al. These additional interventions at index procedures offer some advantages; catheters and the multigate technique, which provides multiple access points [9, 19], are hypothesized to lead to better drainage [9, 17, 49], while coaxial plastic stents and dilation prevent stent occlusion [50, 51] and tract closure [7, 9]. However, LAMS are not designed to work with catheters [11, 52, 53] and repeated tract dilation poses safety risks [9, 41], so these factors may have interfered with how the results may have truly presented in standard practice. Additionally, the evidence supporting the benefits of these additional procedures is also insufficient [9, 11, 49, 54], and they are not part of standard practice, hence, they unnecessarily increased the number of interventions in the trials [7, 9]. This made it difficult to deduce the actual difference in the total number of interventions between the two groups and complicated the overall assessment of stent efficacy in standard practice.

Plastic stents, known for their simplicity and ease of insertion, feature a design with flaps and side holes that facilitate effective drainage. Their most significant advantage over metal stents is their cost‐effectiveness. With a device price tag of under $100, plastic stents offer a far more economical solution compared with expandable metal stents, whose costs can exceed $1800 [55]. This cost‐effectiveness allows for broader access to necessary care for economically constrained environments. LAMS offers a more complex design, incorporating a “barbell” shape with flanged ends for secure anchoring [56]. This advanced integrated configuration, often coupled with the electrocautery‐enhanced delivery system, allows for a single‐step procedure [34], reducing procedure time [56, 57]. Cost analysis revealed that procedural costs were higher for metal stents, but overall cost differences were insignificant. It is likely that the use of coaxial stents and the multigate technique in the metal stents group in some studies [9, 17] may have disproportionately increased procedural costs [22]. Although metal stent devices are more expensive, they constitute only a small fraction of total treatment costs, and the greater number of interventions in the plastic cohort, such as the need for dilation, may balance the costs over time [7, 10]. Metal stents, therefore, may be particularly cost‐effective in treating larger WON, where plastic stents are slower, require additional procedures, and thus lead to higher overall costs.

Adverse events (AEs) include outcomes such as bleeding, stent migration, and death, which we analyzed separately. Trials failed to reach definitive conclusions about these AEs in both metal and plastic groups as well as the optimal stent removal time for metal stents. Bang et al. reported bleeding and other AEs if stent removal exceeded 3 weeks, hypothesizing that LAMS resolve WON more quickly but, being immobile, may damage the cavity's vascular wall [17]. However, other trials [10, 11] suggested that removal at 4 or even 6 weeks might be safe, corroborated by other studies [58, 59]. Our analysis found no significant differences in the various AEs (bleeding, stent migration, and death), but the varying follow‐up and stent removal times across studies make it difficult to determine an optimal stent removal time. WON also has an intrinsic bleeding risk regardless of stent choice or removal [41, 60], and bleeding tends to occur early due to factors such as procedural technique, LAMS’ proximity to vessels, and patient selection and preparation [61]. Therefore, a thorough and careful approach early on is advised, regardless of removal time. Differences in bleeding criteria across studies also led to unstandardized interpretations of outcomes. Follow‐up times in some studies may also not be long enough to detect adverse events [12, 62]. Ultimately, there is a need for standardization in trials to draw more robust conclusions and formulate proper guidelines.

LAMS have demonstrated procedural advantages, but their efficacy in resolving the complex challenges posed by symptomatic WON remains inconclusive [17]. Rigorous investigation into LAMS is crucial to fully assess their potential benefits and limitations. Clinical decision‐making regarding stents should consider patient‐specific factors, as the extent of necrosis in symptomatic WON is closely linked to the type of treatment, including the quantity of solid debris and the number of sessions the patient undergoes [63]. Patients with critical illness who cannot tolerate prolonged procedures, or those in settings lacking fluoroscopic expertise or technical proficiency for plastic stent placement, may be particularly suitable candidates for LAMS, especially if they have predominantly solid WON debris (> 40%) [22, 64]. However, cost considerations and the potential for adverse events, particularly in patients with pseudoaneurysms near WON, must be carefully weighed [17]. To better understand the relative effectiveness and safety of various LAMS designs, further clinical trials are needed to evaluate their unique advantages and potential drawbacks. Additionally, future trials should aim to establish standardized outcomes and procedural techniques to ensure consistency and comparability across studies. This will enable more robust evidence‐based decision‐making and facilitate the development of clear clinical guidelines.

The present meta‐analysis is subject to several limitations. First, there was a lot of variability in outcome definitions such as variations in treatment protocols, including irrigation equipment, catheters, and surgical techniques. The inconsistent definition of clinical success and the diversity of patient populations, including age and disease severity, further complicated comparisons. Second, although the amount of solid debris impacts stent efficacy, inconsistent and variable reporting across studies prevented us from effectively including it in our analysis to assess its impact on outcomes. Third, the inconsistent use of LAMS sizes across studies and within studies limited the ability to conduct meaningful subgroup analyses. Fourth, the inclusion of a study [12] employing both LAMS and BFMS without specifying patient allocation exacerbated heterogeneity. Finally, the asymmetrical distribution of funnel plots indicates potential publication bias, likely due to the preferential publication of studies with positive results. This may have overestimated the efficacy of one stent type. While we included all available data and performed sensitivity analyses, this limitation highlights the need for further studies, including unpublished data, to confirm these findings.

## 5 Conclusion

Despite the larger sample size and hypothesized superiority of metal stents, particularly LAMS, our meta‐analysis did not establish their superiority over plastic stents. While metal stents exhibited lower procedural times and costs, and a trend toward higher short‐term clinical success, other outcomes were comparable. However, we identified substantial heterogeneity among included studies, which may explain the absence of a clear advantage for either stent type. Presently, our study emphasizes the necessity of a tailored, individualized approach rather than a generalized one, focusing on patient‐specific factors such as financial status, chronic illness, and available expertise. Future trials, along with standardization in procedures, analyses, and methodologies are needed to definitively determine the optimal stent choice for WON resolution.

## Ethics Statement

Ethical approval was not required for this meta‐analysis.

## Conflicts of Interest

The authors declare no conflicts of interest.

## Supporting information


**Data S1.** Supporting Information.
