# Supplementary material for: Efficacy of Metal Stents Versus Plastic Stents for Treatment of Walled‐Off Pancreatic Necrosis: A Systematic Review and Meta‐Analysis
Source: JGH Open. 2025 Feb 3;9(2):e70109. doi: 10.1002/jgh3.70109 (PMC11788585; doi:10.1002/jgh3.70109)

**Supplementary file**

**Online supplementary material 1:** Search strategy, PRISMA flowchart and Risk of Bias Assessment

**Online supplementary material 2:** Sensitivity Analysis plots

**Online supplementary material 3:** Funnel plots

**Online table S1:** Detailed search strategy used in each database

| **Database** | **String** | **Results** |
| --- | --- | --- |
| PubMed | ((Walled-Off Necrosis) OR (WON) OR (Pancreatic Necrosis) OR (Necrotizing Pancreatitis)) AND ((Metal Stent*) OR (Lumen Opposing Metal Stent*) OR (LAMS) OR (Electrocautery-Enhanced Stent*) OR (EUS-guided Drainage) OR (Endoscopic Ultrasound-guided Drainage) OR (EUS Drainage)) AND ((Plastic Stent*) OR (Endoscopic Drainage) OR (Percutaneous Drainage)) | 21 |
| Cochrane | ((Walled-Off Necrosis) OR (WON) OR (Pancreatic Necrosis) OR (Necrotizing Pancreatitis)) AND ((Metal Stent*) OR (Lumen-Apposing Metal Stent*) OR (LAMS) OR (Electrocautery-Enhanced Stent*) OR (EUS-Guided Drainage) OR (Endoscopic Ultrasound-Guided Drainage) OR (EUS Drainage)) AND ((Plastic Stent*) OR (Endoscopic Drainage) OR (Percutaneous Drainage) OR (Transmural Drainage) OR (Cystoenterostomy) OR (Necrosectomy) OR (Pancreatic Fluid Collection)) | 108 |

**Online Figure S1:** PRISMA flowchart:


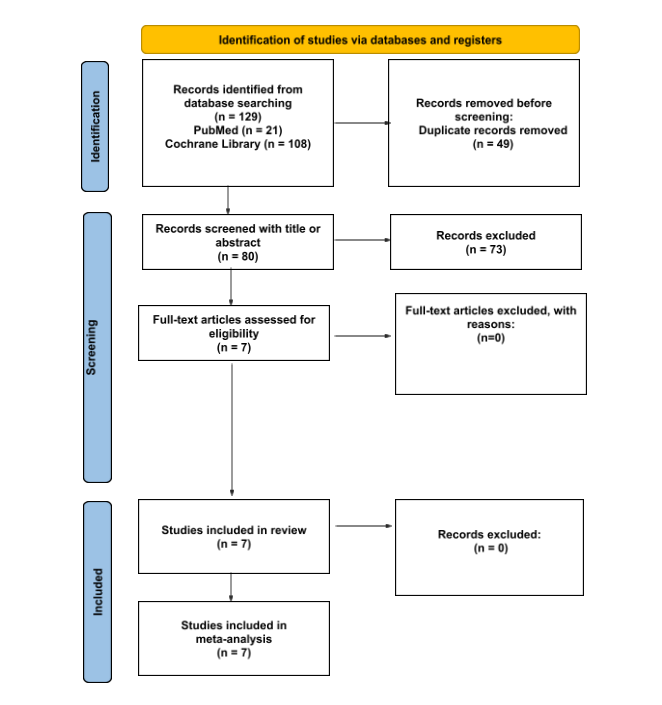


**Online Figure S2:** Cochrane Risk of Bias Assessment of Included Randomized Controlled Trails


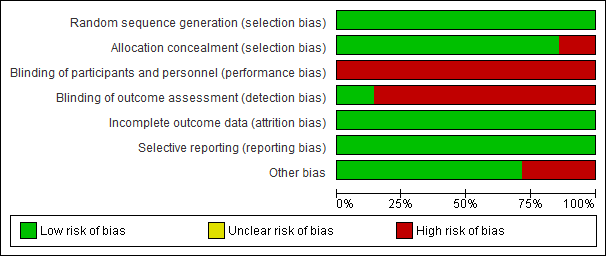


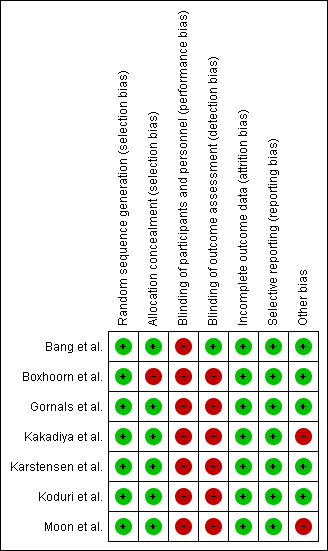


**Online supplementary material 2:** Sensitivity Analysis plots

**Online Figure S3:** Total procedure time sensitivity analysis plot.

**
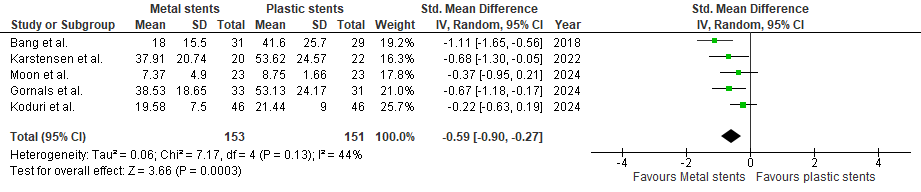
**

**Online Figure S4:** Total procedure cost sensitivity analysis plot.


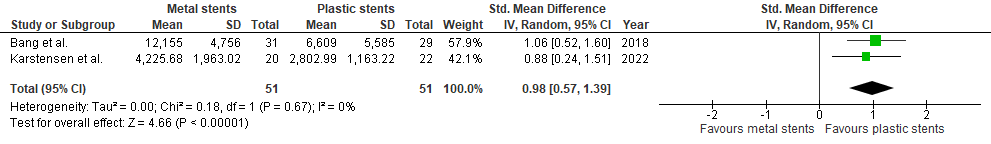


**Online Figure S5:** Hospital stay sensitivity analysis plot.


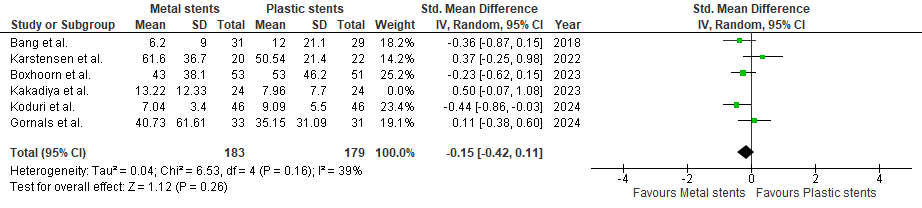


**Online Figure S6:** Total number of necrosectomy sessions sensitivity analysis plot.


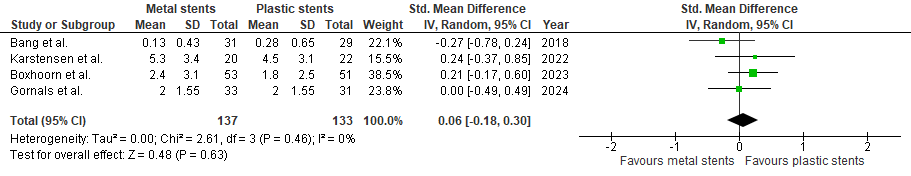


**Online Figure S7:** Need for percutaneous catheter drainage sensitivity analysis plot.

**
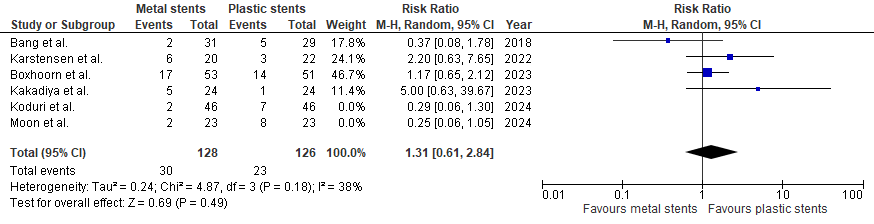
**

**Online supplementary material 3:** Funnel plots

Hospital stay Total procedure time


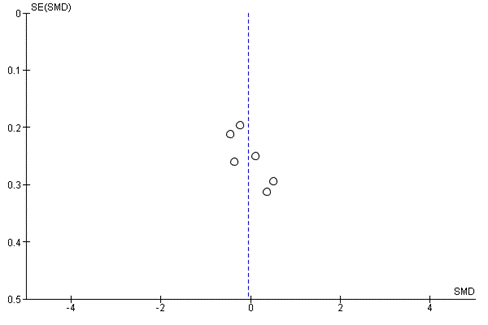

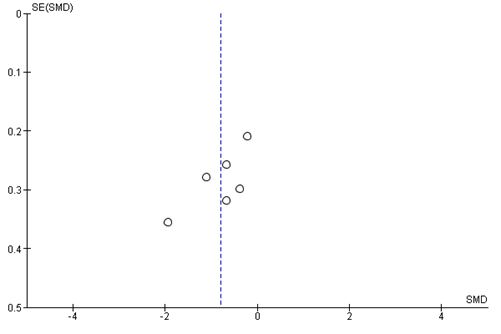


Technical success Clinical success


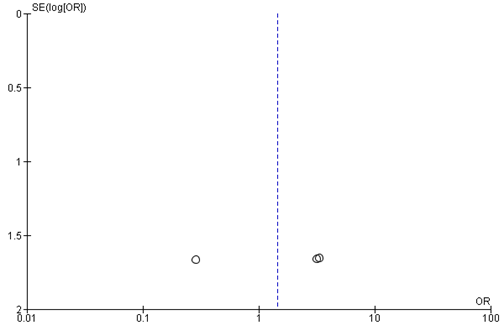

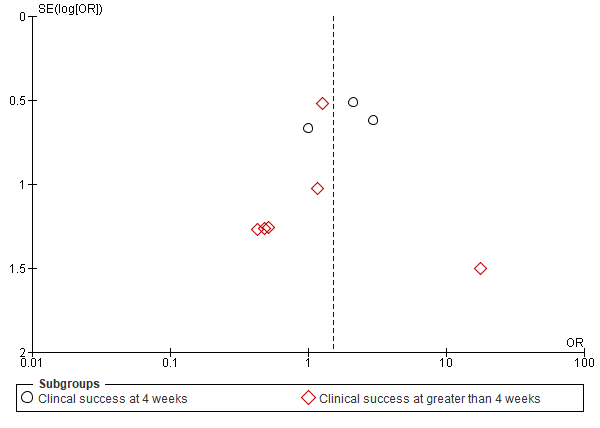


Total number of interventions


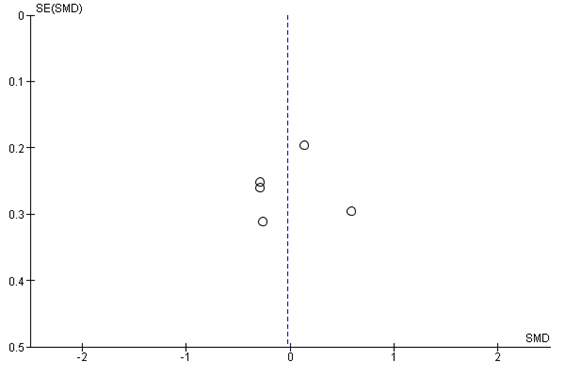


Need for Direct Endoscopy Necrosectomy Total number of necrosectomy sessions


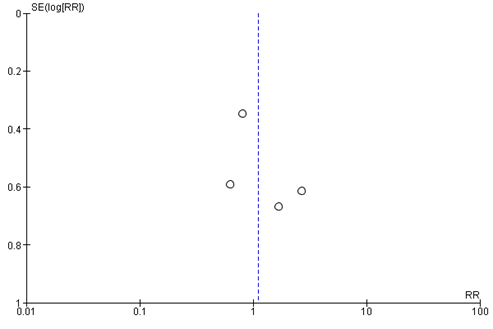

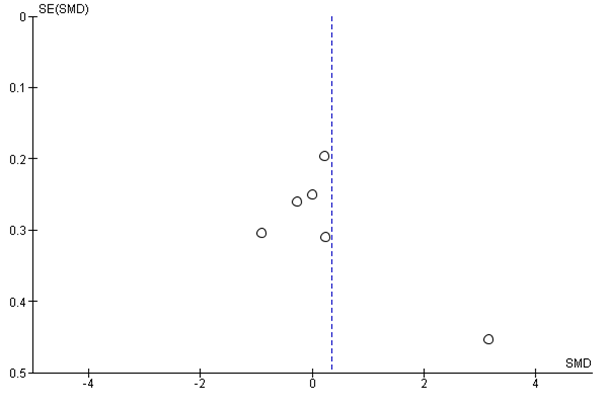


Need for percutaneous catheter drainage Stent migration


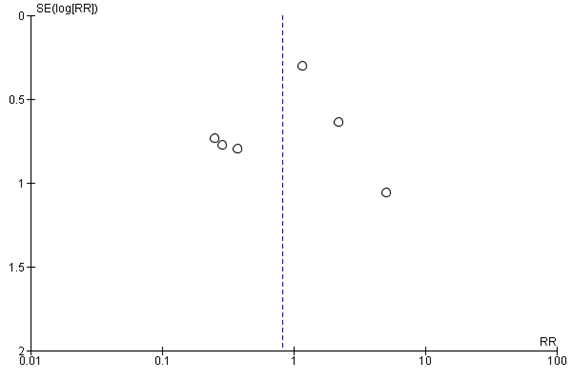

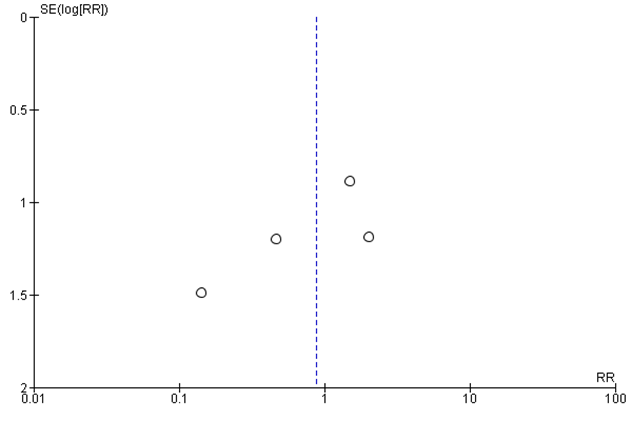


Bleeding Recurrence of Walled off necrosis


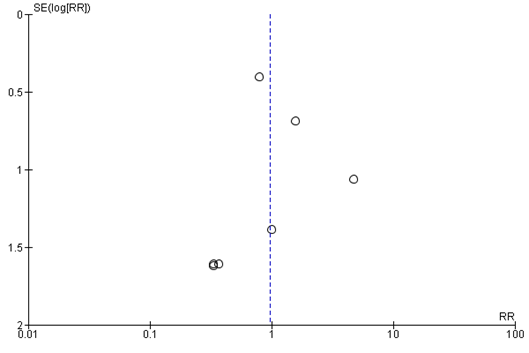

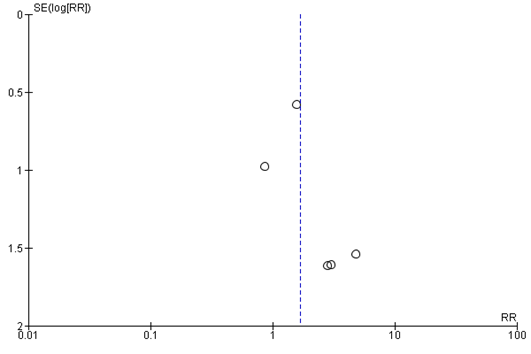


Treatment failure (death) Disconnected pancreatic duct


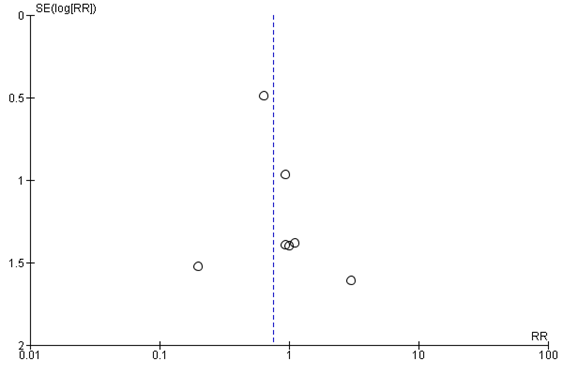

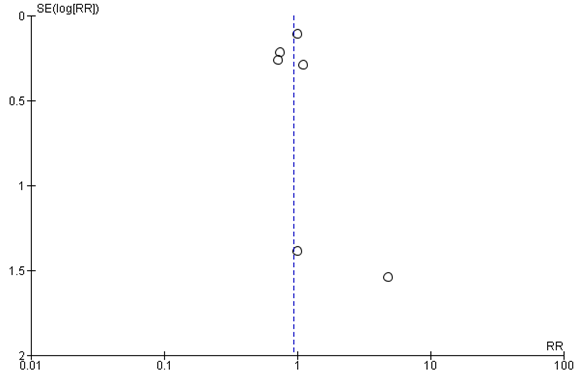


Total cost Procedure cost


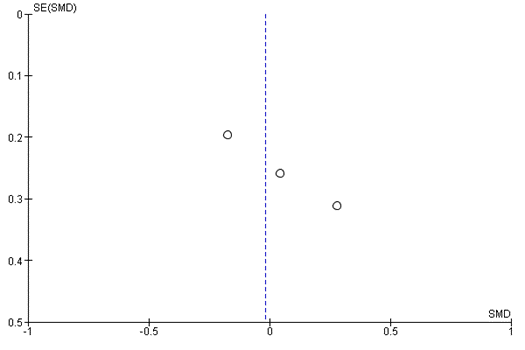

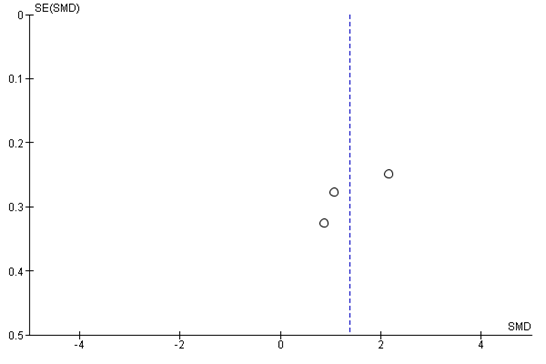

Supplement: Supplementary file 1 — Data S1. Supporting Information. [file JGH3-9-e70109-s001.docx]
